# Illuminating health aspects for immigrant Thai women in Swedish transnational marriages

**DOI:** 10.1186/s12905-024-03071-6

**Published:** 2024-06-12

**Authors:** Weerati Pongthippat, Gunnel Östlund, Mehrdad Darvishpour, Jureerat Kijsomporn, Lena-Karin Gustafsson

**Affiliations:** 1 School of Health, Care and Social Welfare, Mälardalen University, Box 325, Eskilstuna, 631 05 Sweden; 2 Mental Health and Psychiatric Nursing Department, Boromarajonani College of Nursing, Udon Thani, Thailand; 3 Nursing Faculty, Shinawatra University, Bang Toei, Thailand

**Keywords:** Critical incident technique, International marriages, Women’s health, Well-being

## Abstract

**Background:**

Women who are migrants experience discrimination and face major risks, including sexual exploitation, trafficking, and violence, which affect their health and well-being.

This study explored critical health incidents experienced by immigrant Thai women in marriage migration.

**Methods:**

A qualitative explorative approach with in-depth interviews was used. Forty immigrant Thai women who currently or previously had a Swedish spouse were recruited for the study. An inductive critical incident technique was used to collect and analyze the data as the first step. In a second deductive step, the Newman system model was used to categorize health dilemmas.

**Results:**

The women reported 438 critical health incidents in five main areas. Psychological health dilemmas included emotional abuse, feeling overwhelmed due to family responsibilities and the stress of leaving family behind. Sociocultural health dilemmas included transnational family duties or not performing family duties. Physiological health dilemmas included experiencing physical violence and environmental, domestic or work accidents. Developmental health dilemmas included failing health, difficulties upholding the duties expected of a spouse in the target culture and caring for an elderly husband. Spiritual health dilemmas included critical incidents in which the women perceived themselves to have failed in their hopes and duties as a wife, which intensified their dependence on faith, particularly the Buddhist concept of karma.

**Conclusion:**

Professionals in health and welfare practices in Thailand together with professionals in Western countries who work with women in marriage migration situations need to recognize the psychological, sociocultural, physiological, developmental, and spiritual health dilemmas experienced by these women. Furthermore, civil organizations that meet Thai women in foreign countries, such as Buddhist cultural associations, would benefit from the multicultural knowledge revealed by the present study. This knowledge can facilitate healthcare and welfare support for women in marriage migration situations.

## Background

Globalization has increased the trend of marriage migration, which is an old and well-recognized technique to extend the economic resources of individuals and families [1–5]. This feminization of migration, with a higher percentage of female immigrants, is also recognized by gendered patterns of human trafficking and the global inequity between men and women. Women experience a much higher degree of violence in the family situation, which directly affects their health [6]. The process of marriage migration is often problematic because it includes continued economic poverty and increased psychological and physiological health risks [7–11]. Unfortunately, women’s goals to increase the family’s wealth and health are not always met [10]. For example, Thai women who experience spousal intimate partner violence (IPV) in Thailand and leave these relationships through marriage migration seem to form new relationships with Western men who also commit IPV [8]. Moving to another country also involves acculturative stressors while acclimatizing to the new country, including a lack of knowledge of the new culture, a lack of skills in the new language, and increased difficulties with finances [9, 11]. This acculturative stress can be penetrating and prolonged, increasing the risk of emotional and mental health problems [12–14].

Moreover, migrant women’s stress and trauma have been shown to persist in their new culture [13, 15]. Given that migrants experience premigration, migration and postmigration resettlement, the new country’s acceptance of immigrants affects the prevalence of their multidimensional health problems [16]. In Sweden, there is an official stance that gender equality and individual rights, rather than family rights, are publicly prioritized, and these official norms lead to the view that unequal marriages are failures [17].

Marriage migration has also been discussed as a welfare problem based on the marginalized and unequal status experienced by these women [18, 19]. Traditionally, Thai women’s values center on family responsibilities and in-migrant marriages. A key concern is how women manage to handle these responsibilities while also being aware of and caring for their own health needs [17]. Traditions and beliefs in Sweden are very different from those in Thailand. Sweden is a secularized country with Christianity as its traditional belief system, while in Thailand, most inhabitants are Buddhists with a combination of Thai traditions [17]. These public opinions have health consequences for how Thai women navigate marriage migration and how they perceive public reception of themselves and their husbands since the well-being of men is seldom affected by international marriages [4, 10, 17].

Thai women have migrated to Sweden for marriage since 1970. In 2009, there were 17099 Thai migrants living in Sweden, more than 90% of whom were women. Ten years later (in 2019), this number was 43,556 in a total Swedish population of 10 million. The number of Thai female migrants has increased continuously, but in 2021, the gendered proportions changed so that of 44,339 Thai migrants, 9,598 were men [20–22]. Currently, becoming a Swedish citizen through marriage or partnership is more complicated than in the past [23, 24]. However, when the participants of the present study moved to Sweden, they were allowed to become Swedes with equal rights to health care, education, and social services in the new country after two years of cohabitation. According to Swedish law, a cohabitating couple has almost the same rights as a married couple; therefore, the words marriage and cohabitating are used interchangeably in the present paper.

### The aim

A more direct explorative study is needed to empirically extend knowledge of ‘transnational marriage migration’, as research concerning how women cope with immigrant life under these specific circumstances is rare. Therefore, this study explores critical health incidents in everyday life as experienced by Thai women who migrate for marriage.

### Theoretical influence

The study uses an inductive approach, although the NSM [25] inspired our identification of the main health dilemmas or critical incidents in the analysis of the results. Betty Neuman [26] argues that wellness is based on and can be equated to harmony in an individual’s total health profile. Thus, the five domains of wellness include physiological, psychological, sociocultural, developmental, and spiritual variables. Being in a state of “balance” requires harmony among the following variables: the physiological health variable, which refers to the structure and functions of the body; the psychological variable, which refers to mental processes and relationships; the sociocultural variable, which refers to system functions that relate to social and cultural expectations and activities; the developmental variable, which refers to processes related to personal development throughout the lifetime, such as getting older; and the spiritual health variable, which refers to the influence of spiritual beliefs [26, 27]. In the deductive part of our data analysis, we used the five domains of wellness when categorizing the so-called health dilemmas that we identify in our critical incident analysis. However, our examples illustrate that the categorization is not discrete and that intersections exist among the domains because many dilemmas are complex and involve multiple domains.

## Methods

### Study design and participants

A qualitative design with semi-structured questions was used based on the critical incident technique (CIT) [28], which was described by Fridlund et al. [29]. This methodology has the purpose of exploring difficult situations that an individual experiences, so-called critical incidents [30]. The sample included forty immigrant Thai women using the following criteria: (a) women born in Thailand who self-identified as Thai, (b) women who had lived in Sweden for at least five years, and (c) women who were currently or previously married or in a relationship with a Swedish man. The sociodemographic characteristics of the participants are presented in Table 1.

**Table 1:** Socioeconomic and educational characteristics of immigrant Thai women in Sweden (*n* = 40) from interviews in 2016

Interviewees’ characteristics	*N* = 40	
Age at the time of the interview (years)	Min–Max: 27–77	Mean: 51.68
Age upon arrival in Sweden (years)	Min–Max: 17–58	Mean: 33.55
Length of time in Sweden (years)	Min–Max: 6–43	Mean: 18.13
Marital Status		
Cohabitating	27^a^	
Living apart	7	
Divorced	5	
Widowed	1	
Education Level		
Illiterate	5	
Some primary school (4 years)	3	
Primary school (6 years)	9	
Junior high school (9 years)	2	
High school (12 years)	8	
Some college (college)	6	
Bachelor’s degree (university)	7	
Economic Responsibilities in Thailand		
Sends money every month	15	
Sends money on occasion	9	
No duties and no need to send money	6	

^a^Married (19), cohabitating (8)

### Setting and sample

The first author asked three Thai cultural associations located in three differently sized municipalities in southern Sweden to list members who might agree to take part in the research. From the resulting list, sixty women received an invitation letter describing the research project, enquiring about their willingness to participate, and outlining their right to terminate their voluntary participation in the study at any time. A total of 44 participants replied to the first author and confirmed their enrollment as volunteers by telephone,

letter, or email. Ultimately, forty Thai women joined the research project and four women rejected participation, one of whom was forbidden to participate by her partner. Two women felt too anxious and uncomfortable to share their experiences, and one woman missed the interview appointment four times.

The semi-structured interview guide was first pilot tested to appraise functionality, with small corrections made as suggested by Green [31]. The semi-structured interviews were conducted in Thai, Thai and English, or Isan (a local Thai dialect spoken in northeastern Thailand) according to the participant’s preference. The interview started with the question, “Can you describe the last time in your everyday life, preferably in the past week, when you were reminded of being a ‘*mia farang*?’ [Western wife]. The participants were encouraged to provide or expand on details of critical incidents experienced as a Western wife, also known as a *mia farang* in Thai. The interviews were audiotaped and ranged from 50 to 90 min. They were transcribed verbatim in Thai by the first author. The first author and the third coauthor were native Thai speakers. The first three interviews were translated into English by a translator and discussed for preliminary analysis in the research group. The other interviews were preliminarily analyzed by the first author, and these preliminary analyses were presented in English and then discussed by the research group. Validation of the critical health incidents described as part of the interview contents was performed by the Thai bilingual author.

### Data analysis

During the first step of the analysis, the transcribed data were read and the critical incidents were marked in the transcribed text in a process known as vertical reading to compare the critical incidents collected in each specific interview. Then, the interviews were read horizontally to compare the different types of critical incidents collected in all interviews. Contextual and complementary information was encountered through vertical reading, while the horizontal reading was completed to capture common features in the transcribed interviews. The transcripts were then used throughout the analytical process. While preparing the manuscript, the researchers sometimes returned to the interview data. During data reduction, the first author annotated the identified critical health incidents. In the initial part of the analysis, performed using an inductive approach, the critical health incidents were given sticky tags. The analysis then shifted to a more deductive form, and the tags were transferred onto cards extracted from the text to be clustered and organized into groups of health dilemmas guided by the theoretical influence of the Neuman System Model (NSM).

### Ethical considerations

Ethical considerations were necessary during every step of the research process. The interviewer was a mental health nurse educator and Thai woman who completed her PhD in Sweden. She had substantial experience in nursing and counselling. Potential participants were asked by Thai cultural associations in Sweden if they wanted to take part in the research. Women who indicated positive interest were approached by the interviewer, who provided more extensive information about the present project, including information about how the participants’ identities would be kept safe, their right to read the stored data about themselves, which would be kept at the university for 10 years, and their right to withdraw participation at any time. If the women were still prepared to take part in the interviews, a date and time were scheduled. This project was approved by the Uppsala Ethical Vetting Board, number 2016/542.

## Results

As part of everyday life in marriage migration, the immigrant Thai women reported 438 critical health incidents clustered into psychological, sociocultural, physiological, developmental, and spiritual health dilemmas (Table 2). Of the 40 participants, 21 experienced critical incidents that affected their health, while 19 interview participants had experienced incidents in previous periods of migration but were content with their life situation at the time of the interview.

**Table 2:** The main health dilemmas based on categories of critical health incidents derived from interviews with 40 Thai immigrant women in Sweden in 2016

Main health dilemmas	Critical health incidents	Number of women
Psychological health (178 critical health incidents)	A. Emotional abuse and betrayal	14
	B. Abandonment of Thai family members	11
	C. Used as a servant	7
	D. Transferring gambling addiction	6
Sociocultural health (106 critical health incidents)	E. Mothering demands	16
	F. Transnational duties	22
	G. Withdrawal from Thai duties	21
	H. Human trafficking	6
Physiological health (54 critical health incidents)	I. Physical violence	8
	J. Environmental accidents	12
	K. Domestic work accidents	11
	L. Workplace-based accidents	11
Developmental health (44 critical health incidents)	M. Being unable to uphold duties	14
	N. Being left alone	9
Spiritual health (56 critical health incidents)	O. Upholding karma	21

### Psychological health dilemmas

The critical incidents included in psychological health dilemmas were divided into four groups: A. emotional abuse and betrayal; B. abandonment of Thai family members; C. used as a servant; D. transferring gambling addiction.

- A. Emotional abuse and betrayal

The participants thought that showing their loss would be bad for their children, family, and friends because they might lose status, social respect, and face. These women often hid and kept quiet about their situation, even to the children involved, which had major effects on the woman’s psychological health. One woman described the emotional betrayal by her Western spouse when he left her:

*My Swedish husband cheated on me. He had an affair with his secretary and stole money. He left me and our two daughters behind and destroyed our family. My life was altered forever after losing him…I was losing everything and didn’t know what to tell my kids. I couldn’t tell people because I would lose face and pride [tearful]. It felt like I was dying (C-19,59 years old, living in Sweden for 34 years).*

The woman above [C-19] was still affected by the betrayal even though it happened 32 years earlier and seemed to remember it as if it had happened recently. Some of the participants did not reveal the truth to their relatives in Thailand and pretended to still be a married and sharing a household with a Western man.

- B. Abandonment of Thai family members

The participants experienced guilt and anxiety when leaving their families behind. Some women left their children with family in Thailand (e.g., grandparents or other relatives).

*“I came to Sweden because of money and, of course, a Swedish guy, my new fresh [husband]. I sometimes feel regret when I look back; I left my kids behind […] I feel very sad when I struggle here and I cannot raise my kids by myself. Money is needed to pay debts and support my family [...]” (C-14, 58 years old, living in Sweden for 34 years).*

The women worried about their children in their home country. Some made it possible for their children to join them in Sweden, but the situation for these Thai children was often difficult regardless of which country they lived in.

*I have been sending money to my relatives for 11 years. […] I have one 13-year-old son in Thailand who I left behind. He refuses to come here to live. He lives with his grandmother [my old mother] and my relatives. It is very difficult to reconnect with him because I have a new family and a new daughter [he is 24]. I feel regret every day because he has been diagnosed with schizophrenia caused by using drugs. [...] (C-22, 60 years old, living in Sweden for 22 years).*

Some Thai women participants explained that many problems could occur for their children, such as ending up in prison and permanent illness (e.g., drug addiction and psychiatric disorders). They habitually sent money to Thailand and visited home as often as possible, but money cannot raise good offspring.

- C. Used as a servant

Several of the participants emphasized that they never experienced peace and relaxation in their lives because they had to work continuously, which affected their emotional well-being. Being used as a servant in their household could be compared to racist treatment when the whole Swedish family, including Swedish children, mothers-in-law and sometimes friends, groomed them without asking for permission. The Thai women explained that their attention to their Thai and Swedish families and care to others led to tension, worries, and stressful situations. In every situation, the woman tried to please their spouse and new family members. This situation of slavery is demonstrated by the following quote:

*Life with my family and work with the cleaning company was difficult and exhausting […]. I cooked, cleaned, washed, and worked for everyone in the house and found it difficult to find the time to sleep and rest. I saw myself as a slave rather than a wife. Sometimes my Swedish husband treated me badly, along with my mother-in-law and [his] two daughters, who hit and abused me [...]” (C-24, 47 years old, living in Sweden for 10 years).*

The participants pointed out they tried to concentrate on their ‘duties’ and be patient in every situation. Being allowed to send money to Thailand and visit their home country as often as possible helped them withstand the situation. The ability to earn enough money to send home led the women to work even harder than they could manage. However, some spouses treated the women with tenderness and supported them with money and care in their transnational responsibilities and struggles in migration.

- D. Transferring gambling addiction

Some of the participants transferred their previous problems to their new country, such as mental health issues, relational problems, addiction problems and/or debts. Some of the participants explained that their gambling problems mentally stressed them. Gambling addiction negatively affected their lives in both Sweden and Thailand. These women had been addicted to gambling since they lived in Thailand, and their addiction destroyed their relationships and led to continuous psychological dilemmas. Although some participants saw no option but to risk their money by gambling in the hope of getting extra money, excessive gambling often resulted in further debts. Gambling problems led to broken relationships but also allowed them to forget about their responsibilities and release stress.

*I might not be a good mother [2 Thai kids in Thailand, a half-Swedish, half-Thai daughter with her second Swedish husband and a half-Swedish, a half-Thai son with her third Swedish husband], but I am quite good at gambling. When I gamble, I forget about everything (laughing). I am most interested in gambling, which I have been doing since I was 10 years old [...] Gambling is a priority for me since the excitement allows me to release stress, which is a pleasant feeling that makes me happy [...]. Sometimes I lose all the money on slot machines and gambling. I do not have any THB (money) to send to my family in Thailand (C-33, 50 years old, living in Sweden for 16 years).*

A participant said,

*I’ve already sent enough money to my family, and I don’t intend to send any money to my sons. If people don’t like me because of that and talk behind my back, well, I don’t care anymore! [contemptuous smile] I work hard and play hard, and I like the excitement I get from gambling (C-37, 53 years old, living in Sweden for 10 years).*

As the interviewed woman noted, her love of gambling also included the loss of pride and feelings of guilt experienced when she was unable to economically support her family in Thailand. Women’s gambling addictions seemed to not be recognized by the welfare or health services in their new country and were seldom understood by their spouses or the Swedish community.

### Sociocultural health dilemmas

The women described four types of critical incidents categorized as part of sociocultural health dilemmas: E. mothering demands, F. transnational duties, G: withdrawal from Thai duties; and H: human trafficking.

- E. Mothering demands

The participants’ sociocultural belief systems made them cling to their responsibilities of caring for their Thai families and children and their Swedish families and stepchildren. The interviewed women reported acting as nurturing mothers. They were supposed to care for everyone and talked about how their Swedish relatives, such as their stepchildren and mothers-in-law and their husbands’ ex-wives, continued to disrupt their lives. The Thai women presented new problems that happened every day that caused them to feel anxious and worried. Some Swedish husbands made problems worse, and being a nurturing mother and servant was an extra challenge due to demands that might come from almost anyone. One interviewee’s account was as follows:

*I have no kids, but I have to take care of 6 of my Swedish husband’s children who have four different mothers […]. I have to work as a teacher who teaches the Thai mother language […]. Being a Western wife, stepmother, and nurturing mother is difficult and not fun. I sometimes get involved with an ex-wife who treats the kids badly […]. Every so often, I walk through my bedroom after work and shut down my brain, doing nothing […] (C-10, 55 years old, living in Sweden for 18 years).*

The caring responsibilities that most participants experienced made them exhausted and were difficult for some of the participants to handle. Some Swedish spouses seem to be middle persons who could not help with or solve problems. Violence from spouses and poor treatment was also a problem for some Thai women and their children.

*My son moved to Sweden when he was 13. He does not have many friends and is not good at studying or the Thai language, Swedish or English. When my Swedish husband was drunk and we were fighting, I got dragged and knocked out. My son wanted to help me, but he couldn’t because I didn’t allow him and said “Stop! It is not your business” My son was punching the wall and crying. (C-31, 50 years old, living in Sweden for 16 years).*

Some Thai women migrated to Sweden with their Thai children and experienced increasing problems. They reported guilt and embarrassment as they were uncertain how to provide a good life for their children.

- F. Transnational duties

When the participants were accused by their Thai relatives of not fulfilling expected economic duties, they described being tired, feeling hurt, upset, or sad, and feeling embarrassed if they had to or decided to disappoint their family of origin. If the women did not provide transnational help, they did not live up to their Thai families’ expectations regarding what they could receive from a Western woman of wealth. In upholding transnational duties (34), these women tried to live up to the ‘mia pharang’ image of being a wealthy woman who could help everyone in their extended family of origin, which takes a large amount of strength.

*Even though I completed a new house in Thailand [for my mother], I could not stop sending money [...] and I still needed to work two jobs because the money had to be sent monthly. I can’t ignore the needs of my beloved family and relatives who need money and all my support […] (C-36, 54 years old, living in Sweden for 15 years).*

This constant work of different kinds affected the women’s health. They repeatedly experienced critical health situations in family life where they hoped to find peace but instead their domestic life included feelings of exhaustion, strain, and fatigue.

- G: Withdrawal from Thai duties

The participants repeated rather similar experiences related to the duties of Thai daughters, being born into poor families, being equal to other Thai people, and caring for a healthy and wealthy family. For the women’s entire lives, they had fantasized about illusory better lives. Some Thai women were embarrassed, had low self-esteem, and felt guilty when they could not meet the needs of their relatives even when they had become madames who were supposed to have welfare that could be shared. One interviewee described this as follows:

*I have been living in Sweden for 10 years. I started working as a cleaning lady and would send money to my sons and their families. Whatever I sent, it was never enough, though. They always asked for more with no thought toward my health or well-being […]. I just felt stupid constantly giving money to them, so I stopped, not caring what they thought about me […]. They needed to start working for themselves […] (C-37, 53 years old, living in Sweden for 10 years).*

The woman quoted above was fed up with her strained situation and the relatives who never stopped asking for money, so she made the situation come to an end despite cultural norms and expectations. Gaining wealth in Sweden was harder than the women and their Thai relatives imagined. The notion in Thailand is that a madame (i.e., a Western wife) has a husband who can support her, but none of the participants had that kind of situation.

- H. Human trafficking

Some of the interviewed women placed too much faith in other Thai contacts in the new country. Exploitation, insecurity, and rage accompanied these critical health situations when some of the women were misused by people from their own country. Trusting the wrong people could cause the woman to be subjected to human trafficking (8 critical incidents) as sexual objects or as servants; often, both roles were involved.

I felt terrified, worried, and embarrassed. They [my Thai friend and her Swedish husband] took my passport and my money. They kept saying I would get a job […]. I worked for them as a maid for two months […]. I was considering myself; am I her friend or a servant? They were a bad Thai-Swedish couple […]. We fought and they hurt me […] (C-21, 55 years old, living in Sweden for 9 years).

A participant stated,

*The Thai woman at my agency confirmed I could get a job in Sweden […] It paid 100,000 THB […] She took my passport. She asked for money. She tried a matchmaker. Many men came to see me.[…] I needed to work and was not interested in a Swedish spouse […] (C-10, 55 years old, living in Sweden for 18 years).*

Another participant was faced with a similar situation:

*I paid my Thai friend and her Swedish husband 200,000 THB to come to Sweden. They took my visa and forced me to work in their house [...] No work, no money, no future [...] (C-28, 45 years old, living in Sweden for 10 years).*

Some of the interviewed women experienced betrayal by Thai people who were supposed to help them, but the so-called help led to opposite and sometimes even worse situations in the new country since the women did not know who to turn to. Putting trust in so-called Thai friends in Sweden who promised opportunities for wealth and marriage or paid work became an even greater violation and can be understood as part of the sociocultural health dilemmas the women experienced.

### Physiological health dilemmas

In the physiological health dilemma category, four types of critical situations were identified: I. physical violence, J. environmental accidents, K. domestic work accidents, and L. workplace-based accidents. These physiological health dilemmas affected the interviewed women’s health and well-being.

- I. Physical violence

Several of the women were victims of physical and sexual intimate partner violence that was often related to their spouse’s high alcohol consumption. One woman’s account was as follows:

*We had problems for many years, constantly arguing. He hit me when he was drunk [...] I did not tell the police or anyone because I was illiterate and I needed his support. Also, I loved him, and I didn’t want to lose my family... I was embarrassed, I was sick and stayed away from people and the doctor. I hid away and healed myself. I was extremely stressed and unable to sleep […] (C-1, 50 years old, living in Sweden for 15 years).*

A participant stated,

*He destroyed my stuff, especially the Buddha shelf. He punched my face, dragged and threw me and my daughter into the heavy snow (C-35, 38 years old, living in Sweden for 8 years).*

*Not only physical and sexual intimate partner violence but also hurting with bad arguments and fighting.*

- J. Environmental accidents

Regarding the responsibility of financially supporting families in both countries (Sweden and Thailand), most participants were convinced by Thai relatives that transnational support was a requirement for them. This economic dependency on the immigrant women forced them to work even though they physically needed rest. The interviewed women described being subjected to unpredictable environmental accidents, such as slipping on ice, that were sometimes due to a lack of familiarity with the weather and climate:

*I hated to go to work. I wanted to hide under a blanket and sleep in my warm bed, but many bills needed to be paid […]. It was dark and cold...I could not see much of the road in heavy snow, and I had a bicycle accident and broke my left knee [...]. I had two months of sick leave […]. I have only two hands to help myself. The cold weather and sickness make me crazy...I feel blue and depressed [...] [sobbing with a sad face] I have to push myself to survive (C-12, 64 years old, living in Sweden for 37 years).*

This woman felt tired and unable to manage the different weather conditions, and no one cared about her health situation.

- K. Domestic work accidents

A few of the women drank too much alcohol and ended up in accidents and fights. Not behaving correctly and not being able to handle their opportunities as Western wives were sources of shame that were kept in silence. The causes of these accidents only increased the women’s physical health dilemmas and hindered relevant health treatment. Accidents and being unable to work increased the economic strain and made it even more difficult for the immigrant women to continue to meet the tripartite expectations of transnational duties, domestic work, and paid employment. The participants reported that the physiological health dilemmas caused by ‘accidents’ sometimes resulted in the women giving up work for an extended time, and some continued being unable to work altogether due to domestic work accidents.

*It was an accident because I am getting old, 77 years old. Since my Swedish partner passed away, I have been living on my own for 3 years. [...] I have done everything by myself [...] I slipped when I was cleaning the apartment and injured my legs and arms. [...] I was on crutches and was unable to walk for weeks [...] (C-30, 77 years old, living in Sweden for 19 years).*

The Thai women participants repeatedly said that after they lost their Swedish spouses, their life in Sweden became difficult. Some of the women were seeking new partners. Often, their elderly age made it difficult to find a new spouse. Some participants sadly explained that both living in Sweden and returning to Thailand were difficult because they were no longer receiving a Swedish pension.

- L. Workplace-based accidents

Most participants worked in blue-collar positions rather than in well-paid work, although some of them had higher education. The physiological critical incidents included accidents at the workplace, which created misery and economic concerns. Some women felt that they were unable to manage their economic and caring obligations toward everyone.

*I was very tired because of my menstrual period […]. It was dark and cold. I did not see a plastic can on the floor, and I hit it and slipped. It was a workplace-based accident […]. My ankle hurt, and my chest hit the floor. I felt pain, and it hurt when I breathed. The orthopedic found that my ankle was dislocated and my right leg was splinted. He put a cast on me…[sick leave for months] (C-26, 40 years old, living in Sweden for 16 years)*

A participant said,

*On that day, I had almost finished my work (as a cleaner). It was dark and cold. Suddenly, it just happened. I felt like a ghost pushed me. I slipped and hit the concrete [...] bled on my knees and hurt my hands and legs (C-16, 58 years old, living in Sweden for 28 years).*

Some of the women used health care services, but others did not because they felt too ashamed and refrained from showing their physiological injuries even to a nurse or physician. The uneducated participants often resorted to their native upbringing instead and tried to heal themselves with Thai herbs. The physiological capacity of each woman was a human resource that was not looked after well by the woman or by anyone else.

### Developmental health dilemmas

The interviewed women described developmental health dilemmas that were included in two groups of critical incidents: M. being unable to uphold duties and N. being left alone.

- M. Being unable to uphold duties

Being able to uphold family duties while becoming older was a general fear, especially if individuals or their family members became unwell:

*I am unable to read or write, so I work as a cleaning lady [...] My Thai son [is autistic], and now I have been diagnosed with cancer, so I’m thinking about what will happen if something happens to me [...] Can my son survive? Can my son live with his Swedish stepfather? I have these questions in my head every night before I fall asleep (C- 20, 54 years old, living in Sweden for 20 years).*

The participants also talked about how power and strength were or might be lost as they aged. Recognizing aging, including health issues and the inability to work due to cancer, heart disease, stroke, hypertension, or diabetes mellitus, made the women fearful. Some of the interviewed women were unable to work due to failing health, which might worsen with age, and were worried about being left alone in old age.

- N. Being left alone

The interviewed women were concerned with how to adapt to the process of ageing, especially concerning menopause. They worried about being able to uphold their sexual duties, the threat of being left by their spouses and the possibility of being replaced by younger women. The interviewees presented their worries about being left alone without a spouse, and living in fear affected their health.

*I am now 59 with diseases [diabetes and hypertension]. After 25 years living here with just basic Swedish language skills and no English, I can’t explain to Swedish health professionals when I get sick. Shameful, isn’t it? [...]. My spouse had a stroke, and he is gone [died]. I have no kids and no relatives, so I live with my karma. Hopefully, I will have a good life in the future [voice full of sadness]*

*(C-38, 59 years old, living in Sweden for 25 years).*

Some of the interviewed women explained that leaving their older spouse was not an option because of gratitude and fear of karmic retribution in the future or in their next life. The Buddhist belief in reincarnation and karma made their existing life expectancy and transnational responsibilities easier to bear.

### Spiritual health dilemmas as actions

As a previous quote indicated, several critical health incidents were handled from a Buddhist viewpoint, such upholding karma in the duties and migrant life of a Western wife. In critical health incidents, worship was given major significance in the form of prayers to lessen previous bad karma.

- O. Upholding karma

Some of the participants interpreted every critical health incident in life as a spiritual dilemma that had to be handled in a Buddhist way to make amends for failures in this and previous lives.

*I went to the Thai temple in town every week. I gave food to the monks and donated money to apologize for my previous karma [...]. I then seemed to receive better sleep and was relieved of the strain from problems that I was confronted with at work, with poor health, family problems, and so on [...]. You know, we are Buddhists [...] it always helps me (C-12, 64 years old, living in Sweden for 37 years).*

Another expression of faith was to talk to a fortune-teller or a monk to handle critical incidents. When spirituality was experienced as the only salvation from life’s difficulties, these critical incidents were interpreted as spiritual health dilemmas. The participants described spiritual and magical resources as giving hope when they were faced with any negative life event, including different kinds of poor health.

I have my parents cremated remains that I put on a high shelf and pay my respects to every night […]. I also have their pictures in a locket that I wear on a necklace every day [shows the locket]. I certainly believe my parents are beside me. They protect me from bad things and empower me to work and earn money […] (C-32, 45 years old, living in Sweden for 9 years).

Some of the interviewed women described their spirituality as helping them to be more patient and stronger and to not give up hope. According to these participants, spirituality also helped when they were suffering and when they experienced difficult situations while living in Sweden.

The women’s spiritual and religious practices were not always accepted by their spouses. For some of the women, their faith led to relational conflicts when they continued to perform spiritual practices in Sweden. Nevertheless, these women explained that spirituality was deeply rooted in their background and included in their efforts to deal with difficult life situations and change bad karma.

## Discussion

The results of the present study have relevance for the research community as well as for health and welfare professionals nationally and internationally and provide information to professionals regarding the life experiences of so-called Western wives. Knowledge of marriage migration based on women’s lived experiences is relevant in healthcare and welfare services during the life course. Moving to a new country might not change a person’s ability to find wealth and health; however, it could challenge a person’s line of resistance and cause them to withdraw their core energy, affecting their psychological, sociocultural, physiological, developmental, and spiritual health (NSM) [25, 27].

These Thai women’s experiences of health dilemmas contribute to multicultural knowledge about women’s challenges and threats in marriage migration. Based on the interviews, five main risks and challenges related to the identified health dilemmas should be clarified. 1. Women risk being treated negatively by others, including being exploited, betrayed and used as a servant, which directly contradicts the expectations and intentions of marriage migration. 2. Few women found wealth for themselves and their families, even though that was the purpose of moving. 3. The migration emotionally moved the women away from their Thai friends and families due to maintaining face and lacking trust in what (Thai and Swedish) people want from them. 4. There is a spiritual struggle related to developing good karma for the future, which is part of the goal for women and is more difficult to obtain in Sweden. 5. Aging is a major difficulty since the women might lose all of their previous competencies (e.g., mothering in a wide sense) and risk being left alone with no one to turn to. 6. The women must earn a living in blue-collar work (despite their educational level) since Sweden has an individual demand for every citizen to be able to provide for themselves.

Based on these dilemmas, the interviewed women experienced intrapersonal and interpersonal stressors as part of their migration and acculturative stress [12–14]. For example, adapting the Thai style of living to Swedish society's demands could be problematic since every woman needed to earn her own salary. The demand on women to be self-supporting is not compatible with the general Thai public idea of finding a Western husband as a key to happiness and wealth. The participants tried to create wellness for both of their families by ‘mothering’ as low-income mothers might do, which also risked increasing their development of emotional and mental health problems [32]. People’s abilities to handle psychological health dilemmas should also be compared with their premigration experiences since previous experiences and the possible prevalence of mental health problems need to be identified [16].

Sociocultural health dilemmas, such as being overloaded with responsibilities to families in both countries and being torn between two cultural realities, added to the acculturative stress. Due to transnational responsibilities and roles, most migrant women wanted to provide for their extended family of origin, which had high expectations of support [17, 33–35].

When physiological health dilemmas required health care services, some of the women consulted relatives and friends instead, as shown in previous research findings [36]. This is understandable since even marriage migrants must concentrate on earning money [17] and often try to maintain the marital relationship for economic and security reasons rather than love, as marriage migration might have promised [4, 34].

The developmental health dilemmas experienced by the participants included loneliness, little social support, and few contacts in Sweden as well as being caregivers for aging Swedish spouses with no other family members to turn to. Western men tend to seek partnerships with women who are approximately fifteen years younger [37]. This age gap was confirmed by the interviewed women in the present study. According to the NSM [25–27], the interviewed women were subject to intrapersonal stressors enhanced by the ageing process, which also was recognizable in the interviewees’ narratives of critical incidents.

Our results indicated that specific spiritual practices, such as worshipping holy things and asking for guidance from a fortune-teller or Buddhist monk, should be included as part of the interviewed women’s health and wellness system. These spiritual and religious practices seemed to be predominantly supportive and gave the women hope when they were faced with negative events. Women’s health and welfare professionals, especially those who work with women in marriage migration, need to recognize how to meet spiritual needs as part of necessary knowledge in the acculturation process. Research has also shown that immigrant women often rely on religious teaching and beliefs as well as consulting others who have similar beliefs instead of seeking modern medical treatment [38–40].

Sociocultural demands also affect individuals’ adaptation, interpretation and reactions to symptoms of illness, including the ability to seek help, emotionally express themselves and communicate effectively, and navigate interactions with their families and health care providers [41]. Thai professional health workers should consider informing young women about the possible negative aspects of marriage migration, because in Thailand, these negative aspects are silenced. The NSM guides how holistic nursing practice can manage this informational and learning process [42]. The interviewed women who had immigrated to Sweden dreamed of better and wealthier lives [10, 17]. The women’s drive to find wellness can be described as a core of energy and line of potential psychological resilience [27] that could be further developed if the women received culturally informed support and education to nurture their core energy in a new country. Furthermore, learning more about the individually based welfare model used in Sweden compared to the collective and family-oriented model in Thailand is necessary for migrants. Health dilemmas based on reported critical health incidents (e.g., intimate partner violence, mothering two families, domestic and environmental accidents, transferred individual problems, aging) have major influences on life. These negative parts of marriage migration were not expected by the migrating women. The support for marriage migration in Thailand contributes to women´s illusions of the potential and future of being wealthy Western wives [10, 42], and a lack of accurate public health information about marriage migration causes further broken dreams and negative health situations [18].

### Methodological considerations/strengths and limitations

When interpreted through the lens of a multicultural research group, the interviewed women’s lived experiences contribute to extended knowledge of the difficulties that women might experience in marriage migration. The interviewed women’s experiences are partly transferable, especially with regard to migrant women from Thailand but also to other women, especially women from third-world countries. The women´s narratives usually included a chain of situations that were interwoven and contributed to poor health. They were categorized into common health dilemmas that immigrant Thai women experienced as Western wives.

Critical incident methodology has been used in a broad sense to include the women’s descriptions of critical health incidents affecting their lives in Sweden. Flanagan’s [24] critical incident methodology aimed to analyze specific situations of subjective experiences. Likewise, the categorization of the data is not discrete since our examples illustrate that there are intersections among the domains and that many dilemmas are complex and involve multiple domains. Systemic perspectives have no clear distinctions between systems; rather, there needs to be a certain space for crossing and interactions between and within systems [43]. The numbers of critical incidents were more than the 100 required for saturation in CIT studies [24, 29, 44, 45]. The results are credible since the critical incidents collected were related to the women’s ongoing experiences, which were often closely related in time. However, some women managed to find wellness through migration. The fact the situations were remembered is an indication of the seriousness of their subjective experiences. The dependability of the sample was high since they varied in age, educational level, and years of immigration.

Conducting interviews and interpreting the results with first-hand knowledge of Thai culture and language made this research possible. The two Thai native speakers included in the research group were particularly valuable and made the operationalization and interpretation of the results more relevant. A limitation was that not all researchers could read all transcribed data; however, this was also sometimes a strength because the researchers had to repeatedly discuss the content of the interviews, including their reactions to the written English text. Although most of the researchers were experienced, none were native English speakers. Together with the participants, the interviewer created a wealth of unusual data that were further analyzed by the multicultural research group. To our knowledge, the critical incidents that Thai migrant women might experience as Western wives have not previously been described.

## Conclusion

The health and welfare practices in Thailand and Western countries that are included in marriage migration practices, along with civil organizations such as Buddhist cultural associations in foreign countries that support immigrants, could benefit from the multicultural knowledge revealed by the present study. This knowledge could facilitate healthcare and welfare support for women in marriage migration situations.

## Data Availability

The dataset for this current study cannot be made available based on ethical considerations and the possible identification of the participants included in the present study.
